# Candidate powdery mildew resistance gene in wheat landrace cultivar Hongyoumai discovered using SLAF and BSR-seq

**DOI:** 10.1186/s12870-022-03448-5

**Published:** 2022-02-23

**Authors:** Junmei Wang, Yahong Li, Fei Xu, Hongxing Xu, Zihang Han, Lulu Liu, Yuli Song

**Affiliations:** 1 Institute of Plant Protection, Henan Academy of Agricultural Sciences; Key Laboratory of Crop Integrated Pest Management of the Southern of North China, Ministry of Agriculture of the People’s Republic of China, Zhengzhou, 450002 China; 2 School of Life Sciences, Henan University, Kaifeng, 475001 China

**Keywords:** Wheat powdery mildew, Resistance gene, Specific-locus amplified fragment sequencing, Bulked segregant analysis, Gene expression

## Abstract

**Background:**

Wheat powdery mildew, caused by *Blumeria graminis* f. sp. *tritici* (Bgt), is an important disease affecting wheat production. Planting resistant cultivars is an effective, safe, and economical method to control the disease. Map construction using next-generation sequencing facilitates gene cloning based on genetic maps and high-throughput gene expression studies. In this study, specific-locus amplified fragment sequencing (SLAF) was used to analyze Huixianhong (female parent), Hongyoumai (male parent) and two bulks (50 homozygous resistant and 50 susceptible F_2:3_ segregating population derived from Huixianhong × Hongyoumai to determine a candidate gene region for resistance to powdery mildew on the long arm of chromosome 7B in wheat landrace Hongyoumai. Gene expressions of candidate regions were obtained using bulked segregant RNA-seq in 10 homozygous resistant and 10 susceptible progeny inoculated by Bgt.. Candidate genes were obtained using homology-based cloning in two parents.

**Results:**

A 12.95 Mb long candidate region in chromosome 7BL was identified, and five blocks in SLAF matched the scaffold of the existing co-segregation marker *Xmp1207*. In the candidate region, 39 differentially expressed genes were identified using RNA-seq, including RGA4 (Wheat_Chr_Trans_newGene_16173)—a disease resistance protein whose expression was upregulated in the resistant pool at 16 h post inoculation with Bgt. Quantitative reverse transcription (qRT)-PCR was used to further verify the expression patterns in Wheat_Chr_Trans_newGene_16173 that were significantly different in the two parents Hongyoumai and Huixianhong. Two RGA4 genes were cloned based on the sequence of Wheat_Chr_Trans_newGene_16173, respectively from two parent and there was one amino acid mutation: S to G in Huixianhong on 510 loci.

**Conclusion:**

The combination of SLAF and BSR-seq methods identified a candidate region of *pmHYM* in the chromosome 7BL of wheat landrace cultivar Hongyoumai. Comparative analysis between the scaffold of co-segregating marker *Xmp1207* and SLAF-seq showed five matching blocks. qRT-PCR showed that only the resistant gene Wheat_Chr_Trans_newGene_16173 was significantly upregulated in the resistant parent Hongyoumai after inoculation with Bgt, and gene cloning revealed a difference in one amino acid between the two parent genes, indicating it was involved in the resistance response and may be the candidate resistance gene *pmHYM*.

## Background

Powdery mildew, caused by the obligate biotrophic fungus *Blumeria graminis* f. sp. *tritici* (Bgt), is an important disease of wheat [1]. Although wheat powdery mildew can be controlled by fungicides, wheat resistance is the safest and most effective means to prevent or slow the spread of the disease. However, co-evolution and the use of single or few *Pm* genes can lead to rapid development of matching virulence genes in the pathogen population, resulting in breakdown of cultivar resistance [2, 3]. Therefore, it is important to discover, identify, and clone powdery mildew resistance (PMR) genes.

Mapping and homologous cloning are the major methods used to discover resistance genes; however, gene mapping is the basic of the work. Before the development of high-throughput sequencing, many types of markers, such as RFLP, RAPD, SCAR, STS, CAPs, AFLP, and SSR were used in gene marker identification. This method involved screening primers and fine mapping, which was time-consuming and tedious, thereby limiting studies on candidate genes. Single nucleotide polymorphism (SNP) refers to a single nucleotide mutation that causes DNA sequence diversity at the genome level. SNP markers are becoming major molecular markers, with high distribution density, good genetic stability, and a diallelic genotype, which is easy to detect through high-throughput automated sequencing. The increasing throughput of next-generation sequencing and de novo and reference-based SNP discovery has been applied to several species and is gradually becoming the major method for functional gene mining [4–6]. SLAF-seq, based on automated methods, has a significant advantage of high throughput screening [7]. It is an important method for excavating genomic SNP and a high-resolution strategy for large-scale de novo SNP discovery and genotyping. This technology has been made available for haplotype mapping, genetic mapping, linkage mapping, and polymorphism mapping. In previous studies, bulk segregant analysis (BSA), a classical method of marker identification in preliminary screening of primers [8–15], selects 6–10 individuals that represent an extreme phenotype. Due to the small sample sizes, markers identified via this method are usually not closely linked. SLAF uses a minimum of 50 individuals to construct a phenotypic pool, which greatly improves the accuracy of the identified candidate region. Large specific markers of any density that exist throughout the whole genome can be developed based on SLAF-seq to implement fine positioning for functional candidate regions. SLAF-seq has been used for fine mapping and screening of candidate genes in many animals and plants [16–19].

At present, *Pm1* ~ *Pm68* have been named as powdery mildew resistance genes, and *Pm1* (*Pm1a-1e*), *Pm2* (*Pm2a-2c*), *Pm3* (*Pm3a-3j*), *Pm4* (*Pm4a-4d*), *Pm5* (*Pm5a-5e*), *Pm8* (*Pm8* and *Pm17*), and *Pm24* (*Pm24a*-*24b*) contain alleles of these resistance genes [20]. However, there has been little progress in whole wheat sequencing because it is difficult to clone genes from polyploid organisms. Until now only a few *Pm* genes have been cloned, including *Pm3* [21], *Pm38/Yr18/Lr34/Sr57* [22], *Pm8* [23], *Pm46/Yr46/Lr67/Sr55* [24], *Pm2* [25], *Pm60* [26], *Pm17* [27], *Pm21* [28, 29], *Pm24* [30], *Pm5e* [31], and *Pm41* [32].

Of all the identified genes, 30 are derived from common wheat and the remainder are from wild wheat relative species. Exogenous resistance sources were usually used in resistance breeding, sometimes good resistance (*R*) genes exist in the far-edge materials and are difficult to incorporate. Wheat landraces in China are the products of natural and artificial selection and have better adaptability and important value in the practice of resistance breeding. Previous studies elucidated *Pm* gene composition in some wheat landraces in China and showed that the resistance to wheat powdery mildew in Mazamai, Xiabbaidongmai, Youbailan, Hongjuanmang, Aiganmangmai, Hongtoumai, Dahongtou, Hongyanglazi, Jiantouhong, and Bensanyuehuang were attributable to a single recessive gene [33–37]. These studies enrich our understanding of wheat landraces.

Wheat landrace Hongyoumai was collected and conserved by the Germplasm Resources Bank of the Wheat Research Center at Henan Academy of Agricultural Sciences. This landrace was identified to be highly resistant to wheat powdery mildew at both the seedling and adult stages [38]. Xu constructed a Chancellor*Hongyoumai F_2_ hybrid cross, and genetic analysis indicated that Hongyoumai carries one dominant resistance gene to powdery mildew [39]. Wang constructed F_2:3_ segregating population derived from Yumai13*Hongyoumai and reported that the resistance of Hongyoumai to the Bgt isolate GY was controlled by one dominant resistance gene, temporarily named *PmHYM,* which was located on chromosome 7BL near SSR markers [40]. Fu constructed F_2:3_ segregating population derived from Huixianhong*Hongyoumai, and genetic analysis indicated that the resistance of Hongyoumai to the isolate E09 was controlled by one recessive gene, named *pmHYM,* and performed fine mapping to identify co-segregating marker *Xmp1207* of gene *pmHYM* using a 90 k SNP chip [41]. Anti-spectral analysis of 19 wheat powdery mildew pathogen isolates showed that *pmHYM* was different from the *Pm5* gene and its reported allele, and it was a new *Pm5* allele or another gene [41]. The virulence frequencies of wheat powdery mildew isolates collected from different areas of Henan for ten years were lower than 40% according to Li [42] and our unpublished data, indicating that it can still be used in breeding for *Pm* resistance. Transcript sequencing was conducted using Yumai13*Hongyoumai F_2:3_ [43]_._ However, no candidate resistance genes in Hongyoumai have been identified yet.

In previous research, we have constructed the hybrid combinations of Huixianhong (female parent) × Hongyoumai (male parent) and done the identification and analysis of the resistance of the susceptible parents Huixianhong, resistant parent Hongyoumai, and F_2:3_ individuals dirived from Huixianhong ×Hongyoumai. In this study, we used the SLAF-seq approach to analyze the susceptible parent Huixianhong, resistant parent Hongyoumai, resistant pools (Rp) of 50 F_2:3_ homolugous resistant progeny, and susceptible pools (Sp) of 50 F_2_ susceptible progeny derived from the Huixianhong*Hongyoumai F_2:3_ population. We identified differentially expressed genes (DEGs) between Rp of 10 F_2:3_ and Sp of 10 F_2_ using RNA-seq. Screening and expression patterns of target genes were analyzed in the two parents Hongyoumai and Huixianhong, using gene clone and qRT-PCR, respectively. The aim was to find the candidate region of gene pmHYM and further discover candidate PMR regions in Hongyoumai.

## Results

### SNPs accessed using SLAF

SLAF-seq was conducted using an Illumina HiSeq 2500 at Biomarker Technologies, and 194.02 Mb reads were obtained. Two indicators of sequencing quality, Q30 value and guanine-cytosine (GC) content, were 88.09 and 43.44%, respectively (Table 1). Evaluation and monitoring of sequencing data from the control *Oryza sativa* was used to validate enzymatic digestion in this term, and 388,044,970 pair-end reads with average comparison efficiency more than 89.08% using wheat genome as a reference were obtained, along with 614,983 SLAF tags with an average depth of 52.18 in parents and 169.11 in mixed pools (Table 2 and Table 3).

**Table 1:** Evaluation of sample sequencing data

Group	Clean-Reads	Clean-Base	Q30 (%)	GC (%)
R_P_	65,635,954	13,127,190,800	88.62	43.41
S_P_	62,631,433	12,526,286,600	86.46	43.58
F	30,128,177	6,025,635,400	89.05	43.29
M	35,626,921	7,125,384,200	88.24	43.49

**Table 2:** Comparison Statistics with Reference Genomes

Group	Total-reads	Mapped (%)	Properly-mapped (%)
R_P_	13,1271,908	90.15	66.88
S_P_	125,262,866	89.08	65.79
F	60,256,354	90.71	66.50
M	71,253,842	89.97	66.45

**Table 3:** Statistics of SLAF tags

Group	SLAF number	Total depth	Average depth
R_P_	542,168	40,619,006	74.9196
S_P_	554,172	38,178,328	68.8926
F	367,122	18,889,272	51.4523
M	413,249	21,866,603	52.9139
Total	614,983	119,553,209	194.4008

**Fig. 1:**
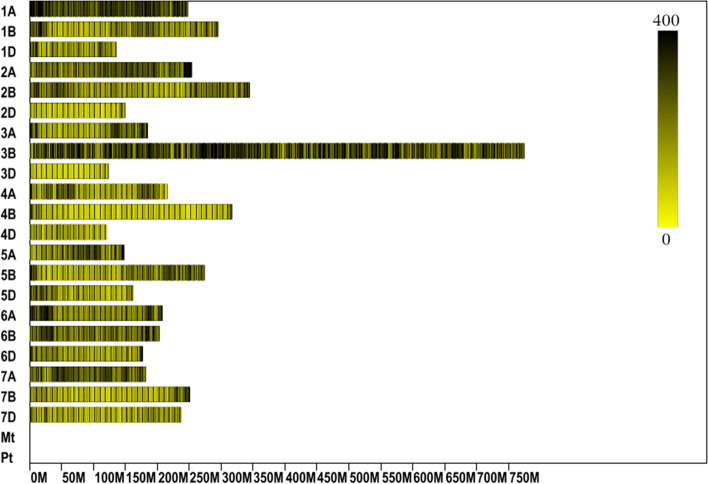
SNP Distribution on wheat Genome

A total of 292,753 SNPs were obtained from two parents, containing 851 non-synonymous-coding regions, as well as 392,556 SNPs in two bulks, containing 1283 non-synonymous-coding regions (Table 4). The distribution of all SNPs on wheat chromosomes is shown in Fig. 1.

**Table 4:** SNP annotation result

Type	F vs M	R_p_ vs S_P_
INTERGENIC	211,552	282,100
INTRAGENIC	2	1
INTRON	1919	2758
UPSTREAM	8730	11,772
DOWNSTREAM	7565	10,735
UTR_5_PRIMER	158	251
UTR_3_PRIMER	313	468
SPLICE_SITE_ACCEPTOR	2	8
SPLICE_SITE_DONOR	6	15
SPLICE_SITE_REGION	52	86
START_GAINED	32	47
START_LOST	0	2
NON-SYNONYMOUS-START	1	0
SYNONYMOUS-CODING	1104	1455
NON-SYNONYMOUS-CODING	851	1283
STOP-GAINED	16	20
STOP-LOST	0	3
Other	60,540	81,552
Total	292,753	392,556

**Fig. 2:**
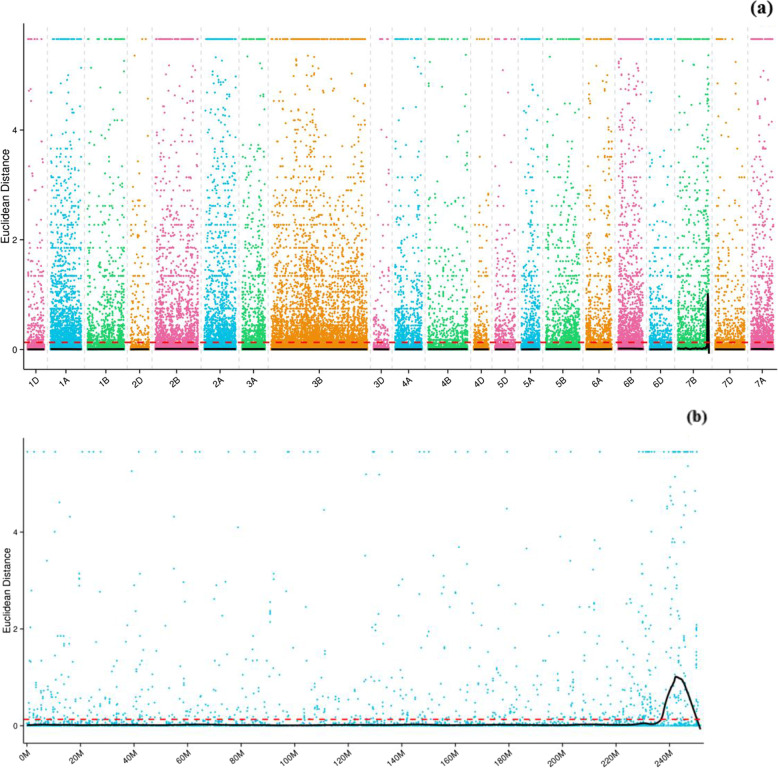
Distribution integration graph of ED correlation values on all chromosomes (**a**) and resolution graph on the chromosome 7B (**b**). Abscissa refers chromosome name;dots represent ED value of every SNP locus;full line represents ED value after fitting; dotted line represents Significant correlation threshold; the higher ED value represent the better the association effect

A total of 210,495 high-quality SNPs were obtained by filtering out loci with multi-alleles, a read support degree < 4, genotype consistency in mixed pools and the parent filtered site. Euclidean distance associate analysis was used to identify a region with a length of 12.95 Mb on chromosome 7BL (Fig. 2 and Table 5).

**Table 5:** Statistical list of associated region information based on ED associate analysis

Chromosome ID	Start	End	Size(Mb)	Gene-Number
7B	237,210,580	250,165,241	12,9547	253

### SNP-index analysis

**Fig. 3:**
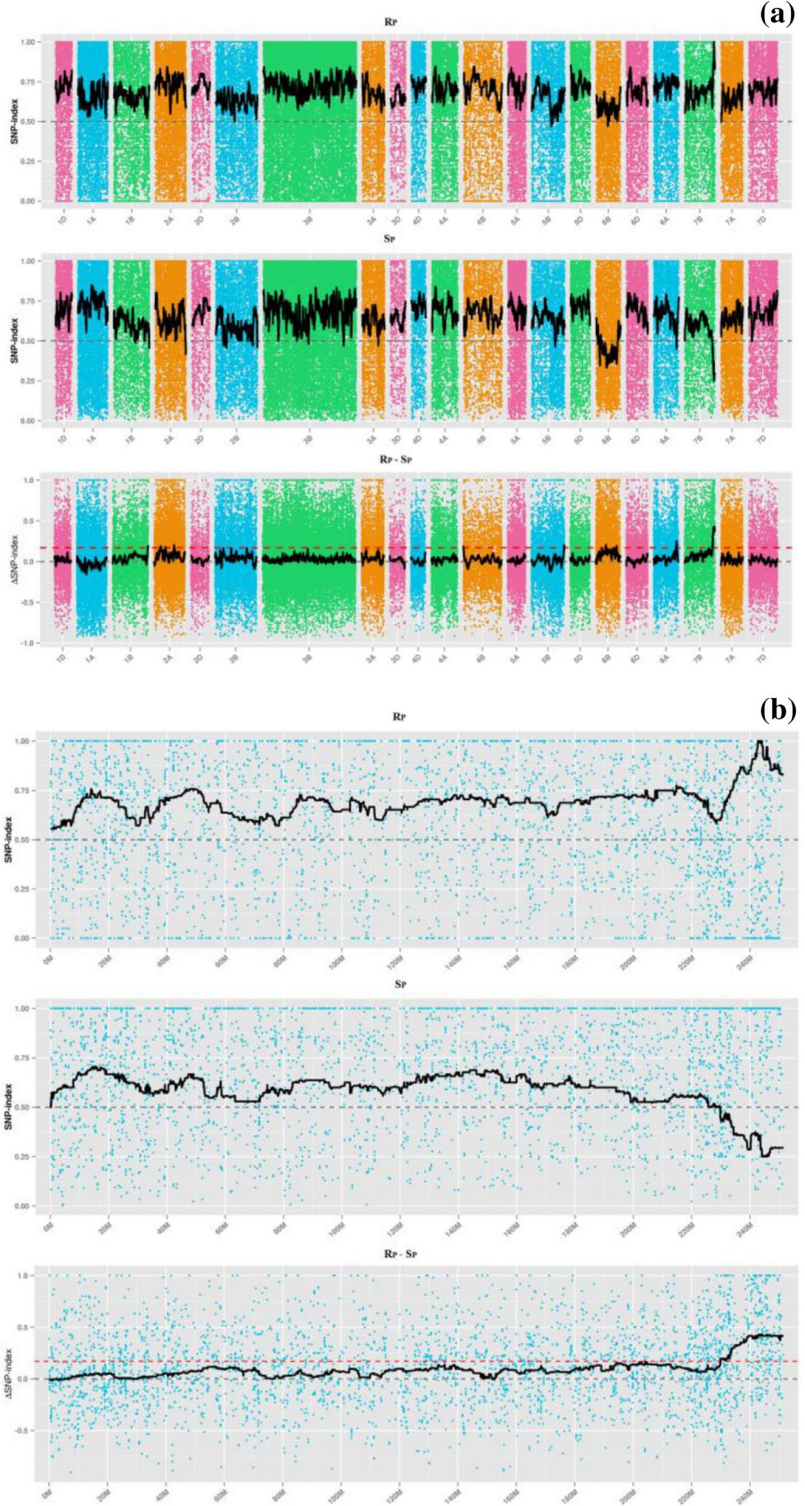
Distribution integration graph of SNP-index correlation values on all chromosomes (**a**) and resolution graph on chromosome 7B (**b**). Abscissa refers chromosome name, color dots represent SNP-index or ΔSNP-index by calculating, black line refers fitting SNP-index or ΔSNP-index. Above figure refers SNP-index value distribution in Rp; middle figure refers SNP-index value distribution in ab pools; bottom figure refers ΔSNP-index value distribution and red line refers theoretical correlation threshold line

SNP-index and ΔSNP-index distribution of the two bulks is shown in Fig. 3. According to separation ratio theory of progeny in the term, the association threshold was 0.667. The region above the threshold was screened and 22 regions were identified, 40.51 Mb in length and containing 649 genes; of these, one was a non-synonymous-coding gene. The region on chromosome 7BL is shown in Table 6. From the intersection of the Euclidean distance and SNP-index, one candidate region, 12.9547 Mb in length, was identified on 7BL from 237,210,580 to 250,165,241, which contained 253 candidate genes. The average distance between the two SLAF markers was 0.0514 cM. The region located in the SSR markers range, corresponding to the region of co-segregating marker *Xmp1207* derived from TA_TGACv1.30.dna.genome|TGACv1_scaffold_578754_7BL was in five blocks: 237371494–241,751,552; 240,463,691–243,321,483; 241,760,892–24,326,939; 241,453,352–243,128,561; 241,721,380–244,424,206.

**Table 6:** Statistical list of associated region information based on SNP-index

Chromosome ID	Start	End	Size(Mb)	Gene-Number
7B	229,913,510	251,503,216	21.59	382

### BSR-seq analysis

#### Sequence assembly and analysis

Over 93 million reads were generated, with 51 million reads from T01 (R-pool) and 42 million from T02 (S-pool). A total of 23.62 Gb clean data were obtained, with a Q30 value of 86.69% and GC content of 55.96% (Table 7). The comparison efficiency of reads from two samples T01 and T02 were 70.59 and 71.76%. A total of 11,233 new genes were excavated and 7887 genes were annotated.

**Table 7:** Evaluation of sample sequencing data by RNA-seq

Sample	BMK-ID	Clean reads	Clean bases	GC Content	% ≥ Q30
R-pool	T01	51,493,576	12,962,382,281	56.62%	86.61%
S-pool	T02	42,324,424	10,657,325,621	55.30%	86.77%

### Differential expression analysis and candidate gene analysis

A total of 1311 DEGs were identified, including 1080 upregulated and 231 downregulated with a Fold-Change ≥2.0 and FDR < 0.01 criterion. Combining SLAF-BSA and RNA-seq, there were 39 known DEGs, including 11 upregulated and 28 downregulated in the candidate region (Table 8). One gene, transcript Wheat_Chr_Trans_newGene_16173, which codes one putative disease resistance protein RGA4, was identified in co-segregating candidate area 243,334,027–243,335,287, and 2^4.67^-fold upregulated genes were expressed in the R-pool than in the S-pool.

**Table 8:** Differential expressed genes in candidate region

Genes #ID	log_2_FC	regulated	nr_annotation
Wheat_Chr_Trans_newGene_16167	6.745021314	up	PREDICTED: polyadenylate-binding protein-interacting protein 8-like [*Brachypodium distachyon*]
Wheat_Chr_Trans_newGene_16173	4.657390482	up	Putative disease resistance protein RGA4 [*Aegilops tauschii*]
Wheat_Chr_Trans_newGene_16408	3.857153144	up	PREDICTED: putative F-box protein At4g17565-like [*Setaria italica*]
gene:Traes_7BL_E41865FE0	2.431156696	up	Putative coatomer subunit beta’-3 [*Aegilops tauschii*]
gene:Traes_7BL_97AC3748A	1.914289176	up	predicted protein [*Hordeum vulgare* subsp. vulgare]
gene:Traes_7BL_418261D09	1.844998764	up	hypothetical protein ZEAMMB73_490100 [*Zea mays*]
gene:Traes_7BL_4437E5002	1.799818185	up	E3 ubiquitin-protein ligase KEG [*Triticum urartu*]
gene:Traes_7BL_A54AA7230	1.594932596	up	hypothetical protein F775_06825 [*Aegilops tauschii*]
gene:Traes_7BL_C09CECD07	1.31525402	up	Transcriptional repressor NF-X1 [*Aegilops tauschii*]
gene:Traes_7BL_E8D5F3A5E	1.159372618	up	predicted protein [*Hordeum vulgare* subsp. vulgare]
gene:Traes_7BL_FD4254327	1.102683172	up	vacuolar proton-inorganic pyrophosphatase [*Hordeum vulgare* subsp. vulgare]
Wheat_Chr_Trans_newGene_16401	−5.91836062	down	–
Wheat_Chr_Trans_newGene_16402	−5.888805499	down	–
Wheat_Chr_Trans_newGene_16164	−5.731202485	down	T-complex protein 1 subunit beta [*Triticum urartu*]
gene:Traes_7BL_95D9D023E	−4.83760605	down	speckle-type POZ protein [*Zea mays*]
gene:Traes_7BL_8B348AE18	−4.746575637	down	predicted protein [*Hordeum vulgare* subsp. vulgare]
gene:Traes_7BL_EA8DE756D	−4.737738698	down	Putative flavin-containing monooxygenase 1 [*Aegilops tauschii*]
Wheat_Chr_Trans_newGene_16411	−4.609251169	down	predicted protein [*Hordeum vulgare* subsp. vulgare]
Wheat_Chr_Trans_newGene_16397	−4.566074614	down	T-complex protein 1 subunit beta [*Triticum urartu*]
Wheat_Chr_Trans_newGene_16399	−4.13389143	down	Speckle-type POZ protein-like protein B [*Aegilops tauschii*]
gene:Traes_7BL_466F701B8	−4.106259873	down	predicted protein [*Hordeum vulgare* subsp. vulgare]
gene:Traes_7BL_AF173EC17	−4.090072315	down	hypothetical protein TRIUR3_06034 [*Triticum urartu*]
gene:Traes_7BL_F45C15FA2	−4.048001723	down	RecName: Full = HMG1/2-like protein [*Triticum aestivum*]
Wheat_Chr_Trans_newGene_16409	−3.873600139	down	hypothetical protein F775_24416 [*Aegilops tauschii*]
gene:Traes_7BL_0FB0AC067	−3.344018347	down	hypothetical protein F775_07814 [*Aegilops tauschii*]
gene:Traes_7BL_AEE3C83EC	−3.090935295	down	predicted protein [*Hordeum vulgare* subsp. vulgare]
gene:Traes_7BL_DE9710474	−3.012246392	down	predicted protein [*Hordeum vulgare* subsp. vulgare]
gene:Traes_7BL_BAFAE0AE2	−2.97032921	down	Protease 2 [*Aegilops tauschii*]
gene:Traes_7BL_B97BBAFAD	−2.867453779	down	PREDICTED: callose synthase 3-like [*Brachypodium distachyon*]
Wheat_Chr_Trans_newGene_16174	−2.647997075	down	–
gene:Traes_7BL_FA7046752	−2.592244631	down	hypothetical protein F775_24157 [*Aegilops tauschii*]
Wheat_Chr_Trans_newGene_16410	−2.565235722	down	Putative disease resistance protein RGA3 [*Aegilops tauschii*]
gene:Traes_7BL_70E7494E5	−2.432573595	down	ent-copalyl diphosphate synthase [*Triticum aestivum*]
gene:Traes_7BL_8A1D0A241	−2.291330273	down	Ent-copalyl diphosphate synthase 1, chloroplastic [*Triticum urartu*]
gene:Traes_7BL_7A3B8A199	−1.975271473	down	RecName: Full = Catalase isozyme 1 [*Hordeum vulgare*]
gene:Traes_7BL_FB212B9AE	−1.960701644	down	oligopeptidase B [*Triticum aestivum*]
gene:Traes_7BL_59EFF59F1	−1.937327117	down	predicted protein [*Hordeum vulgare* subsp. vulgare]
Wheat_Chr_Trans_newGene_16407	−1.92007674	down	–
gene:Traes_7BL_ACE459781	−1.712545481	down	predicted protein [*Hordeum vulgare* subsp. vulgare]

**Fig. 4:**
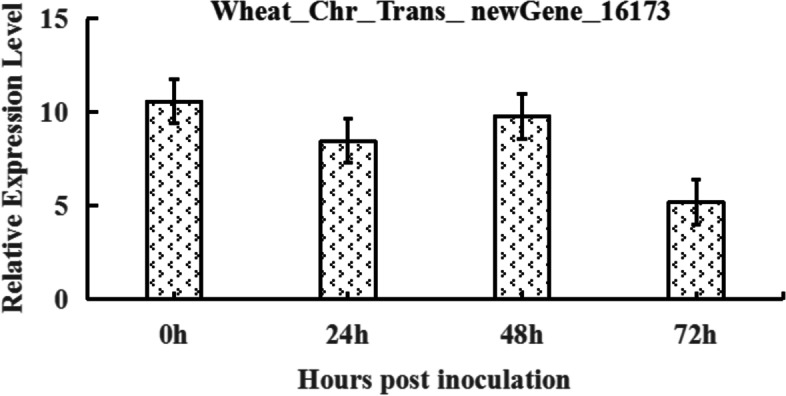
Expression analysis of Wheat_Chr_Trans_newGene_16173. Relative expression level of Wheat_Chr_Trans_newGene_16173 in parent Hongyoumai than Huixianhong at the same time point after inoculation

The expression of Wheat_Chr_Trans_newGene_16173 (gene annotation: Putative disease resistance protein RGA4) in Hongyoumai were 2^3.4^, 2^3.08^, 2^3.29^, 2^2.38^ at 0, 24, 48, 72 h post inoculation (hpi), respectively, which were higher than those in Huixianhong, indicating that it may be involved in the resistance to wheat powdery mildew (Fig. 4). However, no SNPs or InDels in corresponding regions of gene Wheat_Chr_Trans_newGene_16173 were found in the SLAF results, maybe because the sequences in the corresponding region were not identified by SLAF.

### Gene cloning and bioinformation analysis

**Fig. 5:**
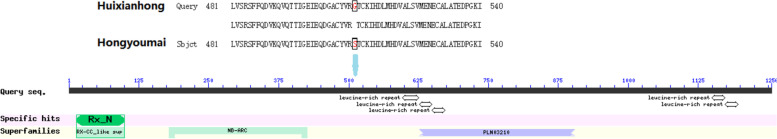
Conserved Structure domain of gene RGA4 and mutation loci in Huixianhong. Bottom areas refers the Conserved Structure domain of gene RGA4; Framed area represents mutation aa

Integrated genes 3780 bp in length were obtained in Huixianhong and Hongyoumai blastx analysis (http://www.ncbi.nlm.nih.gov/), and showed that ORF fragments of Hongyoumai were the same as disease resistance protein RGA4 (Accession no. XM_020324610.1) and one amino acid mutation: S to G in Huixianhong on 510 loci. SMART analysis (http://smart.embl-heidelberg.de/) revealed that coded genes contained seven resistance structure domains: one NB-ARC (179 ~ 463), five leucine-rich repeat (LRR) (597–626; 627–649; 650–673; 1149–1172; 1173–1196), and one LRR_3 (627–940) (Fig. 5).

## Discussion

SLAF is based on SNPs and can identify more abundant polymorphisms than other markers. BSA analysis uses 50 individuals of extreme phenotype. Together they improve the accuracy of mapping and narrow down the candidate regions. An exact resistance region 12.95 Mb in length on chromosome 7BL in wheat landrace Hongyoumai was identified in this study using these methods. Further, five blocks were identified by combining the matching scaffold of co-segregating marker *Xmp1207* and reducing the scope.

Transcriptome sequencing is an efficient method of screening differential expression transcripts, and is a primary method for obtaining some character-related genes combined with subsequent function analysis. In this study, BSR-seq was used to identify possible candidate genes and identified one candidate resistance transcript Wheat_Chr_Trans_newGene_16173, which was differentially expressed in the two parent types, in the co-segregating region. Blastx results showed that the homologous gene of Wheat_Chr_Trans_newGene_16173 belonged to the RGA4-like resistance gene and integrated ORFs were cloned in Hongyoumai and Huixianhong based on reported disease resistance protein RGA4 [*Aegilops tauschii* subsp. *tauschii*] (Accession no. XM_020324610.1).

Resistance genes with LRR domains are widely accepted as playing major roles in plant resistance to pathogens. The LRR domains are thought to interact directly or indirectly with their avirulent effectors to specifically recognize the pathogen. A change in one acid amino in LRR domains may delete or weaken the resistance reaction [44]. Nucleotide-binding site leucine-rich repeat (NBS-LRR) proteins are a current hot topic in plant disease resistance gene research. Genes with NBS-LRR domains are an important part of known resistance genes. NBS conserved domains in resistance genes may have specific relationships with pathogen elicitors and downstream proteins of resistance signal transduct. The NBS domain in NBS-LRR proteins is proposed to function as a molecular switch that adjusts the conformation changes of plant resistance proteins through binding ADP or ATP to regulate resistance signal conduction downstream. There are many NBS-LRR resistance genes and many cloned genes of this kind, including *Pm3* [21], *Pm21* [28, 29], *Pm60* [26], *Pm5e* [31], *Pm41* [32], *Yr10* [44], *Lr1* [45], *Lr10* [46], *Lr21* [47], *Sr33* [48], *Sr35* [49], *Cre1*, and *Cre3* [50]. *Lr24* [51] and *Lr35* [52] are also confirmed NBS genes.

In this study, one wheat powdery mildew resistance gene, a RGA4-like gene, which has one NB-ARC, five LRR domains, and one LRR_3 was identified and cloned and belonged to the typical NBS-LRR-type gene. Also, one amino acid mutation was observed in the susceptible parent Huixianhong and was differentially expressed in two parents*.* This suggests that a RGA4-like gene from Hongyoumai may be the candidate *Pm* gene or one part plays an important role in resistance to wheat powdery mildew. The amino acid mutation locus was outside the conserved domains, the aa change indicated likely presence of another gene or variation in other regulatory elements, so further study is necessary to discover the mechanism of action of RGA4-like gene in Hongyoumai.

## Conclusions

A candidate region for *pmHYM,* 12.95 Mb in length, was located on chromosome 7BL in wheat landrace cultivar Hongyoumai using SLAF-BSA. There were five matching blocks between the scaffold of co-segregating marker *Xmp1207* and SLAF-seq. Using RNA-seq, 11 upregulated and 28 downregulated transcripts were observed in which a disease resistance protein RGA4 (Wheat_Chr_Trans_newGene_16173) was upregulated and expressed in the resistant pool at 16 hpi. Further, qRT-PCR results showed Wheat_Chr_Trans_newGene_16173 was aslo obviously higher expression in Hongyoumai (resistant parent) than in Huixianhong (susceptible parent), indicating it maybe play a role in the resistance response.

## Methods

### Plant materials and treatments

All plant materials, including cultivar Huixianhong (female parent) and wheat landrace Hongyoumai (male parent) F_2:3_ segregating population were derived from Huixianhong × Hongyoumai. Wheat cultivar Jinfeng 1 code was selected and maintained in the wheat disease research laboratory at the Institute of Plant Protection, Henan Academy of Agricultural Sciences, China. The Bgt E09 race was provided by Prof. Yilin Zhou (Institute of Plant Protection, Chinese Academy of Agricultural Sciences).

Huixianhong, Hongyoumai 50 homozygous resistant and 50 susceptible progeny were planted in nutrition pots and placed in a climate chamber at 16–18 °C, with a light/dark photoperiod of 16/8 h, seedlings at the two-leaf and 1 stem stage were collected and mailed to Biomarker Technologies for DNA extraction and SLAF analysis. Two bulk DNA of the F_2:3_ segregating population were developed using Huixianhong as susceptible and Hongyoumai as resistance donors. BSA was conducted on 50 resistant and 50 susceptible progeny.

Ten susceptible progeny and ten F_2:3_ resistant progeny were planted in nutrition pots and maintained in a growth chamber at 16–18 °C, with a light/dark photoperiod of 16/8 h. Inoculation with the Bgt E09 race (maintained on susceptible wheat cultivar Jinfeng 1 code) was performed by shaking conidia onto wheat leaves when plants were at the three-leaf stage. At 16 h post inoculation (hpi), leaves were individually collected, wrapped in tin foil, frozen in liquid nitrogen, and immediately stored at − 80 °C. Then, samples were sent in dry ice to Biomaker Technologies for RNA-sequencing.

Hongyoumai and Huixianhong were planted and inoculated with the Bgt E09 race. The inoculated and control wheat leaves were collected at 0, 24, 48, and 72 hpi, and were immediately frozen in liquid nitrogen and stored at − 80 °C prior to extraction of total RNA.

### SLAF library construction and sequencing

Total genomic DNA was extracted from wheat leaves according to the cetyltrimethylammonium bromide (CTAB) method. DNA quality and concentration were estimated using an ND-1000 spectrophotometer (Nanodrop, Wilmington, DE, USA) and electrophoresis in 0.8% agarose gels with a lambda DNA marker. Wheat genome sequences were constructed as a reference to calculate electronic enzyme digestion using restriction enzymes RsaI. The SLAF library was constructed as described by Sun et al [7], with steps as follows: Genomic DNA of each sample was digested using RsaI and a single nucleotide (A) overhang was added to the digested fragments. Dual-index sequencing adapters were ligated to the A-tailed fragments using T_4_ ligase. Then, the fragments were amplified using PCR and the products were separated using 2.0% agar electrophoresis. Fragments of 364–394 bp in length were excised, purified, and sequenced to obtain target fragments. Pair-end sequencing was conducted using an Illumina HiSeq 2500 system (Illumina, Inc., San Diego, CA, USA) according to the manufacturer’s recommendations (Beijing Biomarker Company).

### SLAF-seq data analysis

SLAF-seq identification and genotyping were performed as described by Sun [7] and Zhang [53]. To assure the quality of sequence analysis, two read lengths of 100 bp were used for data evaluation and analysis. The first 100 bp and last 100 bp in one sequence were both evaluated with a quality score Q30 (indicating a 0.1% chance of error, and 99.9% confidence). Reads <Q30 were filtered out and the ratio of the number of high-quality reads with quality scores >Q30 (indicating a 0.10% chance of error and 99.90% confidence) were added to the total number of raw reads and the GC content was calculated. After the barcodes and the terminal 5 bp positions were trimmed, the high-quality reads were mapped onto the reference genome sequence using SOAP software [54]. Sequences mapping to the same position with over 95% identity were grodowned into one SLAF locus [53]. The sequence error rate was estimated using the *Oryza sativa* genome sequence data as the control. SLAF-tags were identified in parents and mixed pools, and the SLAF-tags that were polymorphic in the female (Hongyoumai) and male (Huixianhong) parents were considered as SLAF markers for subsequent analysis.

### SNP accession and SNP-index analysis

SNP is the major form of polymorphic SLAF. SNP detection was conducted using GATK software and SAM tools [55]. SNPs with common variant loci identified by the two methods were considered valid. SnpEff [55, 56] was used to note and forecast the resulting variation (SNP, Small InDel). Variation regions and effects were identified according to the location and gene information of various loci on the reference genome. High-quality SNPs were obtained as follows. First, SNP loci with many genotypes and reads with a downport degree < 4 bp were filtered out. Second, SNP loci that were coincident between Rp and Sp and genes of Rp did not come from the resistant parent were also filtered out. Third, credible SNPs were obtained. Association analysis was conducted using two methods – Euclidean distance and SNP-index [55, 57, 58]. A candidate region was identified from the intersection of the ED and SNP-index analysis. Genes in the candidate region were annotated using BLAST and the databases NR, Swiss-Prot, GO, KEGG, and COG [57–60].

The ΔSNP-index was calculated using significant differences between bulk genotype frequency. Δ(SNP-index values approaching 1 indicated more SNP markers linked to the target trait.

$$\mathrm{SNP}\_\mathrm{index}\ \left(\mathrm{Rp}\right)=\mathrm{MRp}/\left(\mathrm{PRp}+\mathrm{MRp}\right)$$

$$\mathrm{SNP}\_\mathrm{index}\ \left(\mathrm{Sp}\right)=\mathrm{MSp}/\left(\mathrm{PSp}+\mathrm{MSp}\right)$$

$$\Delta \left(\mathrm{SNP}\_\mathrm{index}\right)=\mathrm{SNP}\_\mathrm{index}\ \left(\mathrm{Rp}\right)-\mathrm{SNP}\_\mathrm{index}\ \left(\mathrm{Sp}\right),$$

where MRp indicates the depth of Rp in the male parent, PRp indicates the depth of Rp in the female parent, MSp indicates the depth of Sp in the male parent, and PSp indicates the depth of Sp in the female parent. False positive loci were filtered out by fitting the ΔSNP-index value of SNP markers from the same chromosome.

### BSR-seq analysis

RNA-seq was conducted using Illumina HiSeq sequencing with two pooled samples of resistance F_2:3_ and susceptible offspring. Each pooled sample contained equal amounts of RNA collected from 10 individuals. Ten resistant plants and 10 susceptible plants were used individually for RNA extracts following the instruction manual of the Trizol Reagent (Life technologies, California, USA). RNA integrity and concentration were checked using a Nanodrop ND-1000 spectrophotometer (Thermo Scientific, Wilmington, DE, USA) and Agilent 2100 Bioanalyzer (Agilent Technologies, Santa Clara, CA, USA). Equal amounts of RNA of qualified samples constituted T01 (R-pool) and T02 (S-pool) for library construction as follows. First, mRNA was isolated using NEBNext Poly (A) mRNA Magnetic Isolation Module (NEB, E7490) and the enriched mRNA was fragmented into approximately 200 nt RNA inserts, which were used to synthesize the first-strand cDNA and the second cDNA. The double-stranded cDNA was end-repaired, de-A-tailed and adaptor ligated. The suitable fragments were isolated using Agencourt AMPure XP beads (Beckman Coulter, Inc.), and enriched using PCR to obtain the cDNA library. Qubit 2.0 and Agilent 2100 were used to detect the concentration and insert size of the cDNA library, respectively, and qPCR was used to detection the exact quantity for effective concentration of the library. Finally, the qualified cDNA libraries were sequenced using an Illumina HiSeq 2500 sequencing platform.

Reads containing adapter sequences, unknown nucleotides > 5%, and low-quality reads of the raw sequencing data, were removed to obtain high-quality clean data (Q30 > 85%). Then, clean reads were mapped to the wheat genome (ftp://ftp.ensemblgenomes.org/pub/plants/release-30/fasta/triticum_aestivum/dna/) using TopHat2. Mapping data, alternative splicing analysis, gene-structure optimization analysis, and the discovery of new genes were performed and gene expression levels were calculated using the reads per kilobase per million mapped reads (RPKM) method. Clean Reads from two samples were used for comparative analysis with the reference genome using TopHat2 Transcripts were filtered for a fold-change threshold of ≥2.0 and ≤ 0.5. The False Discovery Rate (FDR) < 0.01 was applied to identify significantly differentially expressed genes.

### qRT-PCR verification

Total RNA was isolated from wheat leaf extracts using Trizol reagent (TAKARA Biomedical Technology) according to the manufacturer’s instructions. The first strand of cDNA was synthesized from 2 μg of the total RNA using RT-PCR (TAKARA). The cDNA samples were diluted × 10 and used as the templates for qRT-PCR, which was performed on an ABI PRISM 7500 Real-Time PCR System (Applied Biosystems, Foster City, CA, USA). According to the sequence of the candidate gene Wheat_Chr_Trans_newGene_16173 (Putative disease resistance protein RGA4), forwards primer GGGGTCCTCCATCTTC and reverse primer TGGTGCCCAGCCGTT were synthesized to analyze the transcript levels in Hongyoumai and Huixianhong. Wheat 26S rRNA (GenBank accession no. Z11889.1) was used as a control normalization parameter, with primers as follows: 26S-F: GAAGAAGGTCCCAAGGGTTC; 26S-R: TCTCCCTTTAACACCAACGG. The reaction system was as follows: 2 × qPCR Mix 10 μL, 2 μL cDNA, and 1 μL/10 μmol L^− 1^ of each primer, with H_2_O to a total volume of 20 μL. The temperature cycle program was 95 °C for 10 min, 95 °C for 10 s, 55 °C for 30 s, and 72 °C for 34 s for 40 cycles. All reactions were performed in triplicate, including three non-template controls. Dissociation curves were generated for each reaction to ensure specific amplification. Transcript concentration was calculated using 2^-△△Ct^ [61].

### Gene cloning

Primers were synthesized based on the ORF of the sequence of putative disease resistance protein RGA4 [*Aegilops tauschii* subsp. *tauschii*] (Accession no. XM_020324610.1) to amplify full length gene sequences of Hongyoumai and Huixianhong. Forwards primer: 5840-F: ATGGCGGCGACGGT; reverse primer: 5840-R:CTAATCTCTACGGATGGCACATTTCC. Reaction volume: 2 × fast pfu master mix 25 μL, 1 μL 5840-F, 1 μL 5840-R, 3 μL cDNA, with H_2_O up to 50 μL reaction volume. The PCR amplification cycle was 94 °C for 3 min, 94 °C for 30 s, 58 °C for 1 min, 72 °C for 1 kb/30 s for 33 cycles, followed by 72 °C for 10 min. Target gene fragments were isolated using 1.0% agarose gel electrophoresis recovery, purification, and sequencing.

## Abbreviations

Bgt: *Blumeria graminis* f. sp. *tritici*
NGS: Next-Generation Sequencing
SLAF: Specific-Locus Amplified Fragment Sequencing
BSA: Bulked segregant analysis
hpi: hours post inoculation
DEGs: Differential expressed genes
qRT-PCR: Quantitative reverse transcription-Polymerase Chain Reaction
aa: amino acid
PMR: powdery mildew resistance
SNP: Single nucleotide polymorphism,
NBS-LRR: Nucleotide binding site-leucine-rich repeats
GC: guanine-cytosine
ED: Euclidean Distance
RPKM: the reads per kilobase per million mapped reads
FDR: False Discovery Rate
